# Quality of Prison Life and Physical Environment: What Is Predictive of Prisoners’ Overall Satisfaction with the Prison?

**DOI:** 10.3390/healthcare14030299

**Published:** 2026-01-24

**Authors:** Hilde Pape, Berit Johnsen

**Affiliations:** University College of Norwegian Correctional Service, 2001 Lillestrøm, Norway; berit.johnsen@krus.no

**Keywords:** quality of prison life, prisoner–staff relationship, physical environment, cell view, well-being, psychosocial adjustment

## Abstract

**Background/Objectives**: This study examines prisoners’ quality of life by investigating which aspects of imprisonment conditions—including perceptions of the physical environment—best predict overall satisfaction with the prison (OSP). A key question is whether the staff–prisoner relationship is the single most important dimension, which is frequently emphasized in the literature but has scarcely been tested quantitatively. **Methods**: Data stemmed from a survey conducted in three closed prisons in Norway in 2022 (response rate: 63%, n = 163). The dependent variable was assessed by asking: “Generally speaking, on a scale from 1 to 10, how satisfied are you with this prison?” This outcome was regressed on seven subscales from the *Prison Climate Questionnaire* and four single-item measures of the physical environment that have been shown to influence health and well-being. **Results**: As expected, the quality of staff–prisoner relationships had a unique statistical impact on OSP. Ratings of the outdoor areas and the view from the cell were about equally strong predictors. No statistically independent effects were observed for perceived quality of relationships with fellow prisoners, reintegration measures, receiving visits, personal safety, autonomy, access to natural light and a global rating of the prison building (noise, temperature, layout, etc.). **Conclusions**: This study further emphasizes the importance of staff–prisoner relationships in shaping prisoners’ experiences and perceptions of imprisonment. Moreover, it provides new insights into the significance of the physical environment for prisoners’ overall perceptions of prison quality, which is likely to affect their mental health and well-being. These findings have potential implications for the design and siting of new correctional facilities and for improving the quality of existing ones.

## 1. Introduction

Prison climate studies provide insights into prisoners’ perceptions of imprisonment conditions and are typically conceived as an assessment of the quality of life during incarceration [1]. Bosma et al. [2] scrutinized this research literature and identified six primary prison climate domains: social relations, safety and order, contact with people from the outside world, facilities, activities and autonomy. However, according to Ross et al.’s [3] widely cited definition, prison climate also encompasses perceptions of the physical environment. Despite evidence that environmental factors such as proximity to nature, green spaces, and architectural design influence individuals’ health and well-being [4,5,6], these factors have largely been overlooked in research on prisoners’ quality of life.

The potential significance of the physical environment also appears to be largely unrecognized by many prisoners. The following statement from an ex-prisoner vividly illustrates this point while simultaneously highlighting the importance of social relationships [7]:

“Prison life is not easy! Some rules must be followed, and some individuals should be avoided. That’s how it is. […] How interpersonal relationships function is much more important than colour, shape and surroundings! There is not the time nor opportunity to allow environmental trivialities to determine daily life in prison. The most important issues must be confronted: it is the people around you that count”

(pp. 32–33).

Focusing on potential predictors of overall satisfaction with the prison (OSP), the present study of prisoners in Norway examined the impact of a broad range of factors, including aspects of the physical environment that are linked to health and well-being. Drawing on environmental psychology—particularly research on how built and natural environments influence human behaviour and well-being in prison settings—we added questions pertaining to the physical environment to a standardized prison climate questionnaire.

Perceptions of the physical environment may influence prisoners’ experiences of imprisonment independently of the perceived social climate, as they largely operate through different pathways. For instance, pleasant views and proximity to nature can directly affect psychophysiological responses, e.g., [8,9], thereby contributing to subjective prison quality. By contrast, perceptions of non-physical aspects, such as personal safety and relationships with staff, typically develop over time and depend on interpretations and complex psychological processes. Moreover, a prison can have a good social climate despite poor physical conditions, and vice versa. In closed prisons, where social interaction is limited, the immediate physical environment may have a particularly strong influence on individuals’ well-being, independent of the social and relational climate.

### 1.1. The Potential Impact of Relational and Psychosocial Factors on Overall Satisfaction with the Prison (OSP)

How prison officers treat and interact with prisoners, and prisoners’ perceptions of their relationships with staff, are widely recognized as essential to the quality of life during incarceration [1,10,11]. Prison officers hold considerable power, and how they exercise their authority can profoundly shape the everyday lives of prisoners. According to Liebling [12], this relationship constitutes the single most important dimension of prison climate, influencing prisoners’ perceptions of other aspects of life in custody. Similarly, Johnsen and colleagues [13] proposed that the staff–prisoner relationship “determines prisoners’ perception of the quality of prison life to a very significant extent, and that this outweighs other (more material) aspects of prison quality” (p. 523). Moreover, according to a recent review of both quantitative and qualitative research, good staff–prisoner relationships are associated with better quality of life during incarceration, greater prison safety and a more positive overall prison climate [14]. However, whether this relationship is in fact more significant than other aspects of the prison climate has scarcely been tested empirically. The present study of prisoners in Norway adds to this meagre body of research.

Beyond the staff–prisoner relationship, several other factors may significantly influence prisoners’ quality of life. Interpersonal connections are essential for individuals’ mental health and well-being [15], and fellow prisoners most often serve as the primary source of social contact during incarceration. While co-prisoners can provide friendship and emotional support, they can also exacerbate the hardships of imprisonment and present significant health and safety risks [16,17,18]. As briefly reviewed by Bosma et al. [2], a sense of personal safety may also be crucial to prisoners’ overall well-being, along with access to meaningful activities and the extent to which they retain a sense of autonomy.

### 1.2. The Potential Impact of the Physical Environment on OSP

As noted, the physical environment is an integral part of the concept of prison climate and may significantly influence prisoners’ well-being and overall satisfaction with the prison. Environmental stimuli can trigger physiological responses that constitute integral components of emotional states such as stress, tension and anxiety (e.g., [8]). This was recently demonstrated in a study examining prisoners’ and staff reactions to prison design using virtual reality [19]. The physical surroundings—particularly exposure to nature—may also contribute to physiological relaxation and stress reduction [9,20], thereby promoting well-being and conferring health benefits.

Ulrich’s [21] study in a hospital setting has been especially influential in this regard. He found that patients whose rooms overlooked natural scenes exhibited faster postoperative recovery than those whose rooms faced the adjacent buildings. Positive effects have also been observed in studies of prisoners, showing that proximity to nature and access to green spaces are inversely related to rates of self-harm, violent incidents, and emotional distress [22,23,24]. For some prisoners, however, nice views and natural surroundings, as well as glimpses of everyday life in the free world, may serve as poignant reminders of what they are missing [25,26].

Access to natural light and the design of physical spaces are also important environmental factors, influencing mood, mental health and social interactions. Both pleasant views and exposure to natural light can reduce feelings of tension and distress [27], and there is solid evidence that insufficient daylight is associated with an increased risk of depressive symptoms [28]. The physical environment may also affect social interaction and interpersonal relations. For instance, Beijersbergen et al. [29] found that inmates in panopticon-style prisons—where a central observation point allows staff to monitor radiating cell wings from a distance—rated their relationships with staff less positively, suggesting that certain architectural layouts discourage humane staff–prisoner dynamic. Together, such findings underscore the significance of prison siting and design, as emphasized in recent publications on healthy and rehabilitation-promoting living environments for prisoners [25,30,31].

### 1.3. The Norwegian Context

All prisons in Norway are publicly funded, and services such as health care and education are provided through the regular public welfare system—the so-called import model [32,33]. Overcrowding is non-existent, and almost all cells in closed prisons are designed for single occupancy. Each prisoner is assigned a contact officer who is responsible for rehabilitation counselling, and for coordinating external health and social welfare services [34,35].

The so-called Nordic exceptionalism thesis [36,37,38] posits that penal and prison policy in the Nordic countries differs from the punitive approaches in Anglophone countries. According to the thesis, this is reflected in comparatively low incarceration rates, exceptionally humane treatment of prisoners and a strong emphasis on rehabilitation. However, the exceptionalism thesis has been criticized for offering an overly positive portrayal of imprisonment conditions in the Nordic countries. Mathiesen [39], for example, questioned whether psychiatric care and other health services in Norwegian prisons are truly exceptional. In recent years, the prevalence of mental disorders among prisoners in Norway has increased markedly [40], yet the treatment options remain insufficient [41].

### 1.4. Aims

Drawing on data from a Norwegian prison climate study, our aim was to identify predictors of prisoners’ overall satisfaction with the prison (OSP). Given that the staff–prisoner relationship is highlighted as essential to prisoners’ well-being in the research literature, we expected the quality of this relationship to be particularly influential. Owing to the paucity of research, we had no clear expectations regarding the relative impact of either the physical environment or other prison climate dimensions on prisoners’ OSP.

## 2. Materials and Methods

### 2.1. Setting and Locations

In 2022, a mixed-method study on the quality of life in three recently built closed prisons in Norway was conducted [42,43]. The prisons share identical, panopticon-like architecture, and the prisoners have no direct access to the guardroom. Each block contains 96 cells distributed across eight separate wings. Additionally, a small, separate unit accommodates prisoners with severe mental health problems. There are visiting facilities that allow intimate encounters in all three prisons.

The prisons’ perimeter fences are made of chain-link mesh, allowing views of the surroundings. In two of the prisons, the yards are spacious and contain lush vegetation [44]. Both are located on ridges, giving cells views of the yard, nearby forest, and prison buildings. The third prison is in a residential neighbourhood, with some cells facing buffering trees and others overlooking nearby buildings and infrastructure.

### 2.2. The Data Collection

The research team spent 2–3 days in each prison, conducting field work and recruiting participants for the survey. Prior to the data collection, posters containing information about the study were displayed in corridors and other common areas throughout the prisons. Additionally, when approached by the researchers, the prisoners were verbally informed about the study’s aims and the measures taken to safeguard participant privacy and confidentiality. The target sample comprised all inmates in the three prisons, except those housed in the small wings offering special care.

Paper-and-pencil questionnaires, along with written information about the study to ensure informed consent, were available in both Norwegian and English. Additionally, the research team could provide oral translations into Polish and Italian. Most prisoners completed the questionnaires individually, whereas a few did so in small groups with mutual assistance or with the support of a researcher. They could choose where to respond: in the common areas at the wing, in their cell, or in the yard. In total, 181 (62%) prisoners responded, of whom 168 had valid data on the outcome measure and were included in the analyses.

This study is situated within the social sciences, where the principles of the Declaration of Helsinki do not directly apply. However, other regulatory frameworks that largely align with these principles are applicable: the study was conducted in accordance with the Norwegian Research Ethics Act and the Guidelines for Research Ethics in the Social Sciences and the Humanities [45]. It is approved by Sikt (Norwegian Agency for Shared Services in Education and Research) Data Protection Services for research. More information about the data collection is reported elsewhere [42,43].

### 2.3. Measures

The prisoners’ overall satisfaction with the prison (OSP) was assessed by asking; ‘Generally speaking, on a scale from 1 to 10, how satisfied are you with this prison?’ An almost identical question was included in Bosma et al.’s (2020) [2] survey, but they assessed OSP using a 5-point rather than a 10-point response scale. In our questionnaire, the scale endpoints were labelled “very dissatisfied” (coded as 1) and “very satisfied” (coded as 10). Additionally, we applied a dichotomous measure on a high level of satisfaction with the prison, defined as OSP scores of ≥7 (versus lower scores). The OSP question appeared after a series of questions about various aspects of prison life, including those described below. It is therefore likely that the respondents had a broad range of experiences and perceptions related to their imprisonment in mind when answering.

Most of the independent variables stemmed from the *Prison Climate Questionnaire* (PCQ), which is the only validated instrument of its kind [2]. We used PCQ with permission from its developers and translated it into Norwegian without modifying the wording or content. The responses to the items are recoded on a scale ranging from 1 (strongly disagree) to 5 (strongly agree). A sixth response option, “Do not know/not applicable,” was recoded as missing data.

We constructed seven PCQ indices by adding up and averaging the scores on items that captured the following topics: the relationship with fellow prisoners (e.g., “Prisoners here help and support each other”), the staff–prisoner relationship (e.g., “I can talk to staff members in this unit if I feel worried or sad”), autonomy (e.g., “There is much I can decide for myself here”), reintegration (e.g., “In this prison, I can prepare well for my return to society”), activities (e.g., “I am satisfied with the work”), visits (e.g., “The visiting rooms in this institution are pleasant”), and safety (e.g., “I feel unsafe in this prison”). We reverse-scored some items so that higher scores consistently reflected more favourable ratings.

The seven PCQ indices were constructed identically to those used by Bosma et al. [2] and comprise 4–8 items each. Their internal consistency was high, with Cronbach’s alpha ranging from 0.80 (autonomy) to 0.93 (the staff–prisoner relationship). It should be noted that the PCQ covers some additional aspects of the prison climate that our study did not assess (i.e., quality of sleep and care, shop quality, and settlement of complaints).

Four single items captured participants’ ratings of aspects of the physical environment, once again on a scale ranging from 1 (strongly disagree) to 5 (strongly agree). One of these was a stand-alone question in the PCQ about the prison building (“Generally speaking, I am satisfied with the prison building”), with instructions to consider factors such as temperature, fresh air, noise, atmosphere, and layout. The other three items were self-constructed. They focused on the view from the cell (“I like the view from my cell”), access to daylight (“There is enough daylight in this building”), and the outdoor areas (“I like the outdoor areas in this prison”). The first two items were informed by existing literature. The third was included both out of curiosity and in response to the paucity of quantitative measures on the importance of green spaces in prisons in previous research. None of these items were pre-tested.

### 2.4. Statistical Analyses

We examined how the PCQ indices and the single-item measures of the physical environment correlated with prisoners’ overall satisfaction with the prison (OSP), and regressed OSP on these measures in a multiple linear regression analysis. To assess the robustness of the results, sensitivity analyses were conducted. First, we performed logistic regression using the dichotomous measure on a high level of satisfaction (OSP ≥ 7) as outcome. Next, two multiple linear regression analyses were conducted. Specifically, we examined whether the main results persisted when the PCQ dimension related to receiving visitors was excluded, as this variable had a relatively high proportion of missing data—likely because some prisoners never or rarely received visitors. In the second linear regression analysis, we excluded prisoners whose experiences in the current prison were likely limited due to their relatively short stay (<3 months). The data were analysed using SPSS version 31.0.1.0.

## 3. Results

### 3.1. Sample Description

Six in ten respondents (61%) were over 30 years of age, and a similar proportion (59%) were born in Norway or in another Nordic country. Almost three in ten (28%) had no education beyond compulsory schooling, while 15% held an academic degree. The majority had substantial experience with prison life: 70% had been imprisoned for more than a year in their lifetime, and 60% had been in prison more than once. One in four (24%) had been in the current prison for less than three months, while 33% had spent more than a year there.

### 3.2. The Overall Satisfaction with the Prison (OSP) and Its Predictors

The mean score on the 10-point OSP measure was well above the mid-point of the scale (Table 1). Nine of the eleven potential predictors of OSP also had a mean score in the positive range of the scale (i.e., exceeding the neutral value of 3.0), and all correlated positively and significantly with OSP. The perceived quality of the staff–prisoner relationship, the extent of satisfaction with daytime activities, and evaluation of the outdoor areas correlated particularly strongly with OSP (r ≥ 0.60).

Additional analyses showed that nearly all the potential predictors of OSP also correlated significantly with each other (not displayed). This was also the case for the correlations between each of the physical environment variables and each the PCQ indices. The strongest correlations (r ≥ 0.60) were observed between the quality of the staff–prisoner relationship and four of the other PCQ indices. Due to these high intercorrelations, we examined whether including all the potential predictors in multiple regression models would cause multicollinearity problems. The results were reassuring: the highest variance inflation factor was 3.40, and the lowest tolerance estimate was 0.29. Therefore, we proceeded with the planned analyses.

Table 2 displays the results of the multiple linear regression analysis, showing that three of the eleven independent variables had a unique statistical impact on OSP: the quality of the staff–prisoner relationship, and satisfaction with the cell view and the outdoor areas. The strength of their adjusted associations with OSP was similar, with beta coefficients of either 0.24 or 0.26. Altogether, the independent variables accounted for 65% of the variance in the outcome.

### 3.3. Sensitivity Analyses

To examine the robustness of our results, we conducted additional multivariate analyses. First, we replaced the continuous OSP measure with the dichotomous measure of high satisfaction with the prison and performed a logistic regression analysis. The results replicated those shown in Table 2: perceptions of the staff–prisoner relationship, the cell view, and the outdoor areas were the only statistically significant predictors. Additional linear regression analyses yielded the same pattern of results: the main findings persisted when the PCQ dimension related to receiving visitors was excluded and when prisoners with a short stay were omitted.

## 4. Discussion

Consistent with our assumptions, the present study showed that the staff–prisoner relationship had a statistically significant impact on prisoners’ overall satisfaction with the prison. However, the results did not support the widely held view that the quality of this relationship is the single most important factor, outweighing the influence of other aspects of perceived prison quality: features of the physical environment were approximately equally strong predictors of prisoners’ OSP. Our study also showed that the physical environment variables correlated significantly with nearly all the PCQ indices, indicating that these measures capture different aspect of the same underlying construct of prison climate.

There were no statistically independent effects of either perceived quality of relationships with fellow prisoners, reintegration measures, receiving visitors, personal safety, autonomy, or the prisoners’ global rating of the prison building in terms of noise, temperature, layout, etc. However, the additive impact of all the potential predictor variables was considerable, accounting for 65% of the variance in prisoners’ overall satisfaction with the prison.

Prisons have been described as places that epitomize the antithesis of a healthy setting [46]. Indeed, the pains of imprisonment are widely recognized [10,16], and it is well established that imprisonment can significantly compromise health and overall well-being [1,47,48]. However, the respondents in our study tended to hold favourable views about the prison in which they were housed, with a majority reporting high levels of overall satisfaction. This is noteworthy, suggesting that even in closed correctional facilities—with strict regimes and limited freedom of movement—aspects of life in custody may safeguard a sense of well-being. Identifying factors that are predictive of OSP may thus carry important implications for policy and practice.

Predominantly positive views were also observed for nearly all aspects and subdimensions of the prison climate that we assessed. Such results could potentially be interpreted within the framework of the Nordic exceptionalism thesis [36,37,38], reflecting what has been described as exceptionally humane prison conditions in Norway and its neighbouring countries. However, similar findings have also emerged in studies of closed prisons outside the Nordic region [49,50].

### 4.1. The Impact of Staff–Prisoner Relationships and Other PCQ Dimensions

To the best of our knowledge, only one previous study has examined potential predictors of prisoners’ OSP. Drawing on data from a survey of the entire Dutch prison population, Bosma et al. [2] regressed this outcome on all the subscales of the Prison Climate Questionnaire (PCQ), along with several control variables. Our results echoed theirs in important ways. Both studies showed that the staff–prisoner relationship had a unique statistical effect on OSP, and both also indicated that other aspects of the prison climate were approximately equally important. Moreover, somewhat surprisingly, the quality of relationships with fellow prisoners had no independent impact on OSP in either of the two studies.

While some of the other PCQ subscales independently predicted OSP in Bosma et al.’s [2] study, we found that none of them—apart from the staff–prisoner relationship—did. This discrepancy may reflect the fact that Bosma and co-workers assessed only the dimensions captured by the PCQ, whereas ours also included prisoners’ perceptions of the physical environment. The comparability of the two studies may also be questioned for other reasons: whereas inmates from all types of correctional facilities participated in Bosma et al.’s study, our sample was restricted to men incarcerated in recently built closed prisons.

No other studies appear to have tested whether the staff–prisoner relationship is the single most influential factor shaping perceived prison quality, as frequently proposed in the literature, e.g., [12,13]. However, Bosma et al. also regressed a measure on the perceived burden of imprisonment on all PCQ dimensions, and once again, they found no statistically independent effect of the relationship with the staff. Additional analyses of the same dataset similarly showed that the staff–prisoner relationship had no independent impact on either subjective well-being or prisoners’ levels of emotional distress [51].

Taken together, recent survey research suggests that the influence of the relationship with staff might have been somewhat overemphasized in the literature, and that outcomes related to prisoners’ well-being, psychosocial adjustment, and overall imprisonment experience may be more multidetermined than previously assumed. However, the paucity of prison climate studies addressing these issues implies that the evidence base for drawing firm conclusions remains fragile.

### 4.2. The Importance of the Physical Environment

Our multivariate analyses included four single-item measures of the physical environment: prisoners’ perceptions of the prison building (e.g., noise, temperature, layout), access to daylight, the outdoor area, and the cell view. Of these, the latter two both showed a unique statistical effect on OSP. The results further indicated that the perceived quality of the outdoor area and the cell view were both approximately as influential as the staff–prisoner relationship.

The importance of the view may reflect the substantial amount of time inmates in closed prisons typically spend alone in their cells, where the burdens of imprisonment it felt most intensely [43] and visual stimulation is limited. Similarly, as the prison yard is the sole outdoor space accessible, but only for a limited period each day, its perceived quality may account for why it emerged as an important predictor of OSP.

Our findings are consistent with a growing body of research highlighting the significance of proximity to nature and green spaces for the quality of life during imprisonment, which may be even more important for prisoners than for the general population. Indeed, given the inherent burdens and mental health risks of incarceration, humane treatment and prison conditions—including an environment that mitigates these risks and promotes well-being—appear to be of utmost importance.

### 4.3. Methodological Considerations

Research findings from a Norwegian prison climate study cannot readily be generalized to other countries, as implicated by the Nordic exceptionalism thesis [36,37,38] and discussed by Crewe et al. [52] and Brangan [53]. Moreover, nearly four in ten prisoners did not respond to our survey, and it is plausible that non-participation was associated with poor mental health and difficulties adjusting to the prison environment. If so, our study likely presents a more positive impression of the prison climate than is warranted. On the other hand, it is possible that those who were highly discontented with the imprisonment conditions were *more* likely to respond than other prisoners. If the attrition was indeed biased—either positively or negatively—it remains unclear whether, and to what extent, the results of the multivariate analyses were affected.

Focusing on the prison building, the cell view, outdoor areas, and access to daylight, our study offered a glimpse into the prisoners’ perceptions of the physical environment. How they perceived other environmental factors potentially affecting OSP, such as cell facilities and the interior design of indoor common areas, was not assessed. Moreover, OSP was the sole outcome measure, and the robustness of our results would have been strengthened if supported by analyses of additional outcomes.

## 5. Conclusions

Ross and colleagues [3] defined prison climate as a multidimensional concept that encompasses “the social, emotional, organizational, and physical characteristics of a correctional institution as perceived by inmates and staff” (p. 447). The concept thus captures several factors that can potentially influence prisoners’ subjective quality of life. However, although their definition is widely cited in the research literature, it has only been partially applied in quantitative studies, as the perceived physical environment has largely been overlooked. The present study of prisoners’ overall satisfaction with the prison (OSP) strongly suggests that this dimension is important and should be incorporated into standardized tools aimed at assessing the overall prison climate.

Our analyses revealed that three prison-climate dimensions had a particularly strong impact on OSP: the quality of the staff–prisoner relationship, the view from the cell window, and satisfaction with the outdoor areas. These findings may inform the training and education of prison staff, the planning, siting and design of new prisons, and initiatives to enhance the quality of existing facilities.

We applied a composite measure of perceived quality of the staff–prisoner relationship; however, certain aspects of this relationship may be more important than others, and this issue warrants further empirical investigation. Moreover, to gain a deeper understanding of how prisoners perceive their physical surroundings and how these perceptions shape their overall prison experience, we recommend complementing survey research with qualitative approaches. In-depth interviews could elucidate the reasoning underlying survey responses and provide richer, potentially more policy-relevant contextual insights.

## Figures and Tables

**Table 1:** Mean values on prison climate measures, and their zero-order correlations (95% confidence intervals) with the overall satisfaction with the prison (OSP).

	Mean (SD)	Correlations ^1^ (95% CI) with OSP	n
The overall satisfaction with the prison (OSP) ^2^	6.15 (2.60)	---	168
The staff–prisoner relationship ^3^	3.21 (0.99)	0.61 (0.50–0.70)	156
The relationship with co-prisoners ^3^	3.83 (0.76)	0.34 (0.20–0.47)	160
Autonomy ^3^	2.87 (0.98)	0.53 (0.41–0.64)	162
Safety ^3^	3.89 (1.01)	0.37 (0.23–0.50)	158
Activities ^3^	3.18 (0.89)	0.65 (0.55–0.73)	160
Reintegration ^3^	2.72 (1.10)	0.54 (0.46–0.67)	152
Visits ^3^	3.31 (0.78)	0.59 (0.48–0.69)	146
The prison building ^3^	3.21 (1.28)	0.48 (0.34–0.59)	152
The view from the cell ^3^	3.35 (1.23)	0.50 (0.38–0.61)	157
Access to daylight ^3^	3.69 (1.12)	0.50 (0.38–0.62)	159
The outdoor area ^3^	3.65 (1.16)	0.60 (0.49–0.69)	158

^1^ All correlations (Pearson’s r) are statistically significant at the 1‰ level. ^2^ Scale range: 1–10. ^3^ Scale range: 1–5.

**Table 2:** Multiple linear regression analysis showing the unique statistical impact of potential predictors on the prisoner’s overall level of satisfaction with the prison. Variables with a statistically significant impact are printed in bold.

	B	S.E.	Beta	p
Relationship with staff	0.62	0.28	0.24	0.03
Relationship with co-prisoners	0.14	0.24	0.04	0.56
Autonomy	0.08	0.24	0.03	0.75
Safety	0.25	0.17	0.10	0.17
Activities	0.44	0.28	0.15	0.12
Reintegration	0.24	0.20	0.10	0.23
Visits	−0.27	0.32	−0.08	0.41
The prison building	0.14	0.15	0.07	0.36
Access to daylight	0.05	0.19	0.02	0.78
The outdoor area	0.55	0.16	0.26	<0.001
The view from the cell	0.56	0.17	0.26	<0.01
	Adjusted R^2^ = 0.64			

## Data Availability

The dataset presented in this article is not available because of ethical restrictions concerning data on prisoners.
